# Stochastic modeling of economic injury levels with respect to yearly trends in price commodity

**DOI:** 10.1093/jis/14.1.59

**Published:** 2014-01-01

**Authors:** Petros Damos

**Affiliations:** Laboratory of Applied Zoology and Parasitilogy, Department of Crop Production (Field Crops and Ecology,Horticulture and Viticulture, and Plant Protection), Faculty of Agriculture, Forestry and Natural Environment,Aristotle University of Thessaloniki, 541 24 Thessaloniki, Greece

**Keywords:** AR model, crop quality assurance, integrated pest management, plant protection

## Abstract

The economic injury level (EIL) concept integrates economics and biology and uses chemical applications in crop protection only when economic loss by pests is anticipated. The EIL is defined by five primary variables: the cost of management tactic per production unit, the price of commodity, the injury units per pest, the damage per unit injury, and the proportionate reduction of injury averted by the application of a tactic. The above variables are related according to the formula EIL = C/VIDK. The observable dynamic alteration of the EIL due to its different parameters is a major characteristic of its concept. In this study, the yearly effect of the economic variables is assessed, and in particular the influence of the parameter commodity value on the shape of the EIL function. In addition, to predict the effects of the economic variables on the EIL level, yearly commodity values were incorporated in the EIL formula and the generated outcomes were further modelled with stochastic linear autoregressive models having different orders. According to the *AR(1)* model, forecasts for the five-year period of 2010–2015 ranged from 2.33 to 2.41 specimens per sampling unit. These values represent a threshold that is in reasonable limits to justify future control actions. Management actions as related to productivity and price commodity significantly affect costs of crop production and thus define the adoption of IPM and sustainable crop production systems at local and international levels.

## Introduction

The economic injury level (EIL) is an important concept in crop production and agriculture because it quantifies the cost/benefit ratio that underlies all pest control decisions to be adopted in integrated pest management (IPM) and sustainable agriculture. IPM involves coordinated use of multiple tactics for optimizing the control of all classes of pests (insects, pathogens, vertebrates, and weeds) in an ecologically and economically sound manner ( Dickler and Schäfermeyer 1991 ; Dent 1994 ; Altieri and Nicholls 2000 ; Agra CEAS 2002 ; Damos and Savopoulou-Soultani 2012 ).

Concerns about consequences related to the use of non-selective insecticides have increased the interest in the development of alternative means for pest control that have little or no impact on humans, beneficial organisms, and sensitive ecosystems ( Higley and Pedigo 1993 , 1996; Ehler 2006 ; Ifoulis and Savopoulou-Soultani 2006 ). Traditionally, conventional plant protection strategies are associated to a variety of problems, including environmental side effects, insecticide resistance, negative impacts on natural enemies, safety for pesticide applicators, and important implications for the food supply due to unacceptable pesticides residues ( Altieri and Nicholls 2000 ; IPM Europe 2000 ). Moreover, the type of pest management actions, as related to productivity and price commodity, significantly affects the costs of crop production and thus defines the adoption of IPM and sustainable crop production systems at a local or even international level ( Altieri and Nicholls 2000 ; IPM Europe 2000 ).

Lately, the major IPM principles have been outlined by the European Commission and the European Parliament (adopted in the second reading, European Commission 2009a , b). Particularly, eight general principles IPM are currently identified and related to the following topics ( European Commission 2009a , b; Damos and Savopoulou-Soultani 2012 ):

1. Measures for prevention and/or suppression of harmful organisms

2. Tools for monitoring

3. Threshold values as basis for decisionmaking

4. Non-chemical methods to be preferred

5. Target-specificity and minimization of side effects

6. Reduction of use to necessary levels

7. Application of anti-resistance strategies

8. Records, monitoring, documentation and check of success

In practical terms, the major goal of IPM is not to eradicate pest populations but to accept the presence of a tolerable pest density, conserve environmental quality, and improve user profits ( Pedigo et al. 1986 ; Higley and Pedigo 1993 ; Boller et all. 2004 ). However, this approach relies on the development and application of economic injury levels (EIL) and economic (action) thresholds ( Cross and Dickler 1994 ; Buntin et al. 1996 ; Damos and Savopoulou-Soultani 2008 , 2009, 2010).

Generally, for the application of EILs, the development of a mathematical relation between insect pest injury and yield loss needs to be established first. This relation is called “damage function” or “damage curve” and consists of the biological part of the EIL concept ( Buntin et al. 1996 ). If the damage function has been evaluated, the next step is to estimate future projections of the EILs in respect to the economic variables ( Peterson and Hunt 2003 ; Damos and Savopoulou-Soultani 2009 , 2012).

The EIL by definition consists of an empirical relation rather than a dynamic one, and therefore the aim of the current work is not to rediscover that the EIL varies with changing market values of crops. In contrary, using this basic principle, efforts are made to move beyond the traditional cost-benefit equation and to capture the nonlinear trends of EIL, utile for short coming extensions and long term validations, using stochastic models. In this context, the aim of the current work is to develop a general model that describes and predicts the yearly trends of EILs. Based on prior studies, yearly trends of the price commodity in the EIL model are incorporated in order to make future predictions. By keeping the deterministic skeleton of the EIL formula, first a yearly time series is generated in respect of the yearly trends of the price commodity. Finally, a stochastic linear autoregressive model is applied to make predictions for the EIL for the forthcoming years.

### The concept of economic injury levels

The EIL is cornerstone for IPM and crop protection because it defines how much pest injury can be tolerated. The concept of the EIL integrates biology and economics and uses control actions (mostly pesticides) only when economic loss is anticipated (Pedigo et al. 1996). The EIL is further used to define the economic threshold, which is the operational criterion used by plant protection advisors and farmers to define the population density at which control measures should be initiated to prevent an increasing pest population from reaching the EIL (Pedigo et al. 1996).

The EIL is based on the relation of five primary variables and can be estimated according to the following formula EIL *= C/VIDK,* in which *C* represents the cost of management tactic per production unit, *V* is the price of commodity, *I* is the injury units per pest, *D* is the damage per unit of insect injury, and *K* is the proportionate reduction of injury averted by the application of a tactic ( Buntin 1996 ; Damos and Savopoulou-Soultani 2009 , 2012). The variables *I* and *D* are related to each other and are the biological characteristics of the function by representing the yield loss associated per pest. The parameters *D* and *I* can be obtained from the slope of the yield, or damage function ( *Y = a + bx* ), where *Y* = yield loss; *a* = 0, *x* = number of pests per sampling unit; and *b* = yield loss/pest, representing the loss per insect, which is equal to I*D or *D’* ( Damos and Savopoulou-Soultani 2009 ).

In order to model yearly trends of the EIL, a representative industrial peach cultivation was chosen as a case study. In particular, the values of the EIL were estimated during 14 successive years (1996–2010) in respect to the price commodity of each year (as given by public peach corporations and the Greek ministry of rural development and food). Moreover, for simplicity reasons, in this study other variables of the EIL model were considered as constant.

### Stochastic modelling of the economic injury level

To address the challenge of modelling the EIL, a stochastic linear autoregressive model was applied. By using the EIL formula, first a yearly time series based on the parameters of each year was generated ( Damos and Savopoulou-Soultani 2009 ). These yearly values of the EILs result in a stationary stochastic process that can be further represented by a linear autoregressive model with infinite order and uncorrelated residuals.

Thus, considering no density dependency but similar variations, the EIL process equals:

$y_t = \delta_t + \sum_{j=0}^{\infty}\theta_j \epsilon _{t-j}$

$= \delta_t + \epsilon_t +\theta_{1}\epsilon _{t-1} + \theta_{2}\epsilon_{t-2} + L$

$= \delta_t + \epsilon_t +\theta_{1}L\epsilon _{t} + \theta_{2}L^{2}\epsilon_{t} + L$

$= \delta_t + \theta(L)\epsilon_t$

(Equation 1)

where *{s _t_ } ^+^ _2* is a sequence of random variables known as white noise, satisfying that: *E(s _t_ ) =* 0 and *E(e ^2^ ) = o ^2^* with zero auto-covariance in all cases and *S _t_* deterministic component.

Hence, *e _t_ =N(0,o ^2^ ),* and *N* stands for the normal distribution having variance *o ^2^* , and *8 _t_* is a deterministic component that is predictable from its past history and is uncorrelated with *e _t_ _* for all *j* , and *{6* }0°° are square sumable

$\sum_{j=0}^{+\infty}\theta_{j}^{2} < \infty, \text{with}\, \theta_0 = 1$

$\text{Let}\, \theta(L)\approx \sum_{j=0}^{p}\alpha_{j}y_{t-j}$

(Equation 2)

where *L* represents the lag operator, such *t* ha *tL ^j^ x _t_ =x _t_ _j, p=1,2,…,k* is the time lag of the stochastic process, so that in any of the EIL time series v, may be written as:

$y_{t} = u + (\sum_{j=0}^{p}\alpha_{j}y_{t-j})\varepsilon_{t}$

(Equation 3)

or:

$y_{t} = u + a_{j}y_{t-1} + ... + a_{p}y_{t-p} + \varepsilon_{t}$

(Equation 4)

where *e _t_* has zero mean and variance *a ^2^* or *s _t_ = WN(0,o _E_ )* and *u* is assumed to be negligible.

Equation (4) consists of the autoregressive model of order *p* , *AR(p),* in which the constant term *u* satisfies the condition that data do not have a zero mean ( Brockwell and Davis 1996 ; Grunwald et al. 2000 ) .

In the current work, three autoregressive models of order *1* , *2* , and *3* were generated, and their performances were statistically compared.

### Parameter estimation

Parameter estimates of the *AR(p)* were based on prediction error decomposition and maximum likelihood estimates. Considering that the probability of the sequence of the EIL residuals is given by:

$L = \prod_{i=p+1}^{N}p(\varepsilon_i)$

(Equation 5)

which by terms of the joint density function is (Harvey 1993):

$f(y;a,\sigma^2) = f(y_1,...,y_{n-1};a,\sigma^2) = f(y_n\mid y_{n-1},...,y_1;a,\sigma^2)$

(Equation 6)

The rule is further applied for all successive observations obtaining:

$f(y;a,\sigma^2) = \prod_{j=9+1}^{n}f(y_i\mid y_{j-1};a,\sigma^2).f(y_1,...,y_{p};a,\sigma^2)$

(Equation 7)

where *y _j-1_ = (y _1_ ,...,y _j_ - _1_ )ʹ.*

Since each one of the conditional distributions of the *AR* model is:

$N(\sum_{p}^{j=1}a_{j}y_{t-j}, \sigma^2)$

(Equation 8)

and

$f(y_{1},...,y_{p};a,\sigma^2)$

(Equation 9)

is the marginal distribution of the first *p* observations.

The exact log likelihood for the *AR(p)* model is given by (Hamilton 1994):

$l(a,\sigma^2;{y}) = log[L(a,\sigma^2;y)] = -[\frac{(n-p)}{2}]log(2\pi\sigma^2)$

$-\frac{1}{2}\sum_{t=p+1}^{n}(\frac{y_t-\sum_{j=1}^{p}a_{j}y_{t-j})}{\sigma^2} + log[f(y_1,...,y_{p};a,\sigma_{2})$

(Equation 10)

Considering EIL as a time series and in the case of the *AR* having order 1, the relevant marginal distribution is:

$f(y_{1};a,\sigma^2)$

and

$y_{1} \approx N(0,\frac{\sigma^2}{1-a_{1}^{2}})$

(Equation 11),

equation (10) results to:

$$log f(y_1;a_1,\sigma^2) = -\frac{1}{2} \{log(2\pi) + log(\frac {\sigma^2}{1-a_{1}^{2}}) + y_{1}^{2} (\frac {1-a_1}{^2} \sigma ^2)\}$$

(Equation 12).

### Model comparison

In order to determine the effect of more parameters on the *AR* model the Akaike informational criterion *(AIC)* were used ( Akaike 1974 ; Damos and Savopoulou 2010 ). Thus, if *x* is the vector of the time series observations used to estimate the parameters ****6**** of the *m* model among *M* candidate models *(m=1,2,…,M),* then considering that *g(x0 _m_ (x))* is the maximum likelihood function for model *m* and ****p**** is the number of parameters, then the *AIC* is:

$AIC(m) = -2\text{ln}\, g(x\mid \hat{\theta}_m(x))+2\rho $

(Equation 13)

The above criterion permits inferences on how the different number of parameters adds to the explanatory power of the candidate model ( Damos and Savopoulou-Soultani 2011 ).

The short described mathematical framework and the related assumptions and principles were used to analyze the time series structure of the EIL.

## Results and Discussion

**Figure 1.**
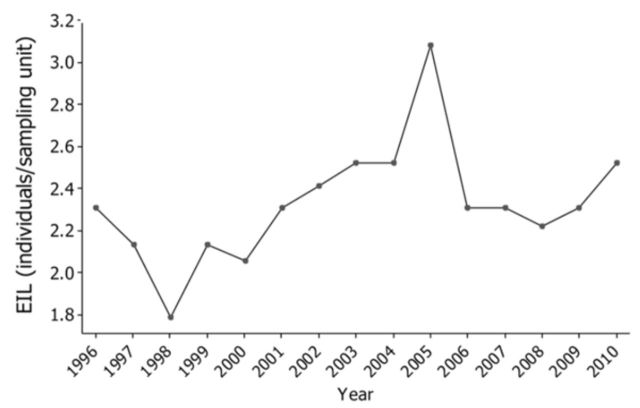
Time series plot of the EIL (insect larvae/fruit) in respect to observation year. High quality figures are available online.

Figure 1 depicts the generated time series of the EILs in respect to year. The plotted EIL values represent the number of individual pests (i.e., moth larvae) detected at each sampling unit (i.e., fruit). These values were estimated in respect to the mean seasonal trends of the price commodity for representative industrial peach varieties of public fruit corporations of Northern Greece and by keeping other parameters constant. The observable dynamic alteration of the EIL is the result of yearly changes on its different parameters (here: price commodity). However, one can not exclude that value of the EILs can be slightly modified with respect to the other economic parameters, such as the mean market price for different pesticide categories.

**Figure 2.**
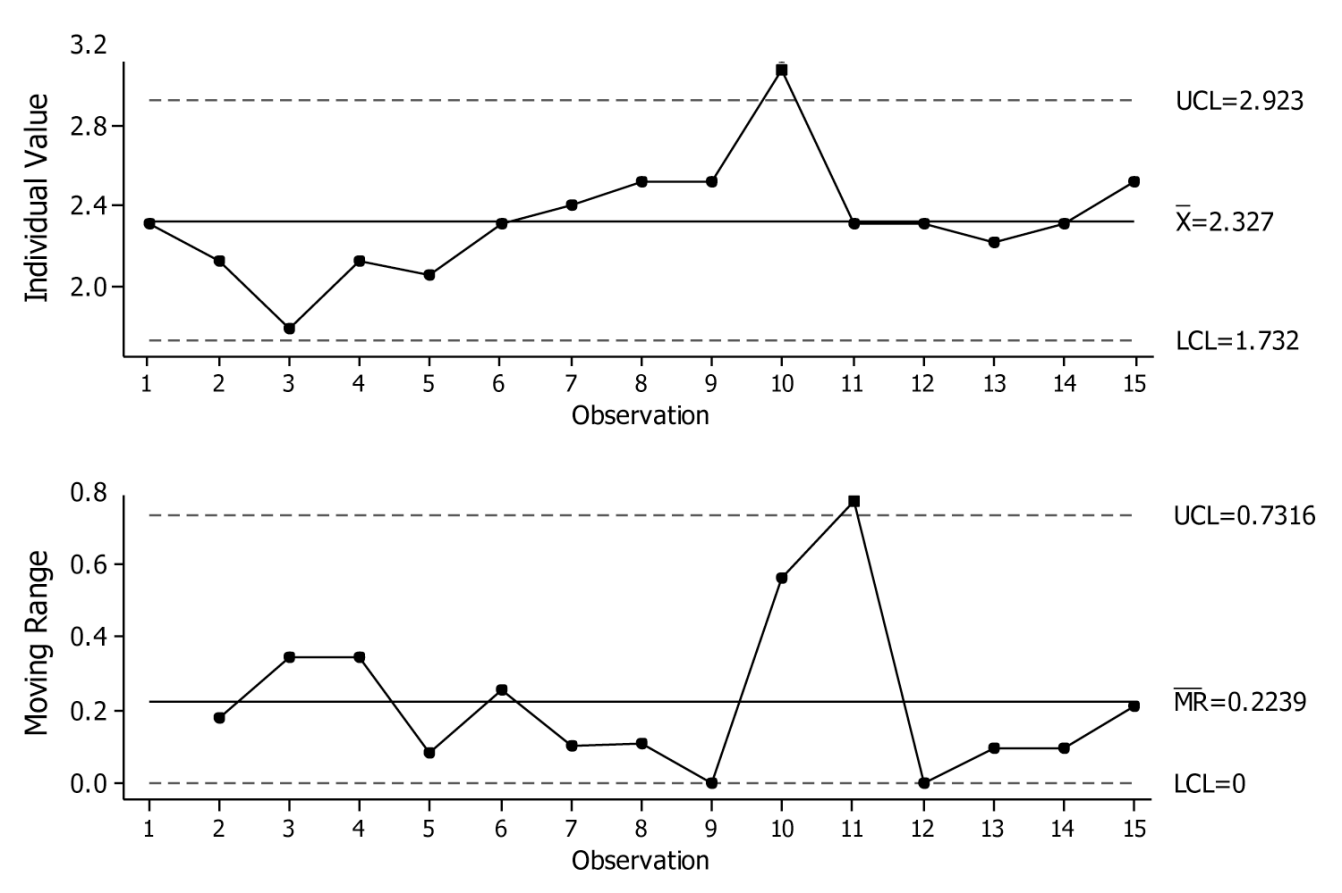
Individual moving range chart that tracks the levels and range of the EIL stochastic process and respective upper (UCL) and lower (LCL) 95% confidence intervals. High quality figures are available online.

Figure 2 displays the individual control charts of the EIL variable of interest in regards to of the successive-yearly observation points. These charts track both the process level and process variation, and at the same time detect outliers. Only one point is more than threefold standard deviations from the center lines. This indicates that almost all observed values of the EIL are normally distributed in their sampling space.

Further more, Figure 2 by default estimates the process variation, *s* with MR/ d2, and the average of the moving range divided by an unbiasing constant. The moving range is of length 2, since consecutive values have the greatest chance of being alike. Special causes result in variation on the EIL that can be detected and, to some extent, be managed, while on the other hand common cause variation is inherent in the process. Hence, the EIL dynamic stochastic process is in control when only common causes (not special causes) affect the process output because most values fall within the bounds of the control limits and do not display non random patterns ( Damos et al. 2011 ).

To date, these values represent how measurements of the EIL samples process may change over time and are practically used to define if the EIL is a weak stationary processes and can be described by an autoregressive model with normally distributed errors.

**Figure 3.**
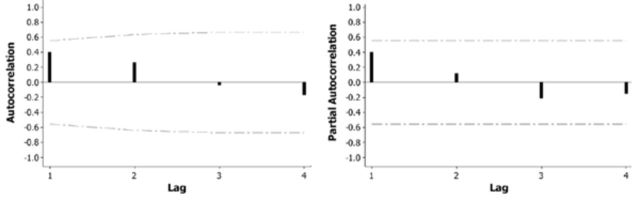
Autocorrelation (left) and partial autocorrelation (right) and respective 5% confidence intervals for the EIL regarded as a time series variable. High quality figures are available online.

Figure 3 depicts the autocorrelation and partial autocorrelation function for the EIL variable. In both cases, the process is dependent on short previous values. This is indicated by the decrease in the correlations of the successive series points separated by *k* time units. The detection of significant time lags is fundamental for the description of any stochastic process (i.e., population feedbacks are fundamental characteristics of ecological organisa-organisation). More important, such kind of information is a prerequisite for further autoregressive modelling. For instance, because autocorrelation and partial autocorrelation drop considerable from lag1 towards lag3, we come to the conclusion that the process has short memory. In other words, the EIL values of the foregoing years exert influence on the EILs of the coming year. Understanding how time lags contribute to the EIL projected values, we are able to detect seasonal trends and periodicity utile in forecasting.

**Figure 5.**
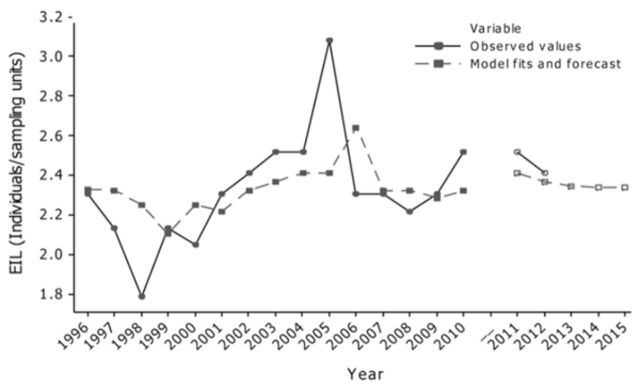
Time series plot of the EIL (insect larvae/fruit) and *AR(1)* model fits in respect to observation year (1996–2010); updated EIL values (2011–2012) and forecast of the *AR(1)* models for the successive five-year period (2011–2015). High quality figures are available online.

Table 1 gives the estimated parameters of the three linear autoregressive models that were applied to describe the EIL as stochastic process. The performance statistics in respect to model order are also given. According to the maximum likelihood estimates and the related AICs, the *AR(1)* model can be used to describe the EIL process because more number of parameters do not add to the explanatory power of the *AR* model. These estimates reveal the physical process that builds persistence in to EIL and are further used to generate future values (i.e., Figure 5 ). Moreover, despite the EIL being conversant at present only, it is actually composed of parameters either uncertain or entirely doubtful, none of which (unfortunately) can be predicted directly. Therefore, this analysis advances the science behind EIL’s current autoregressive approach and takes into account the magnitude of the likelihood that the EIL will take certain values. Thus, if there is a ‘true’ EIL, it should be stochastic rather than deterministic, and from this standpoint the current approach may advance the EIL not only from a descriptive-deterministic representation towards a statistical-stochastic one, but also to a tool used to perform predictions.

**Table 1.**
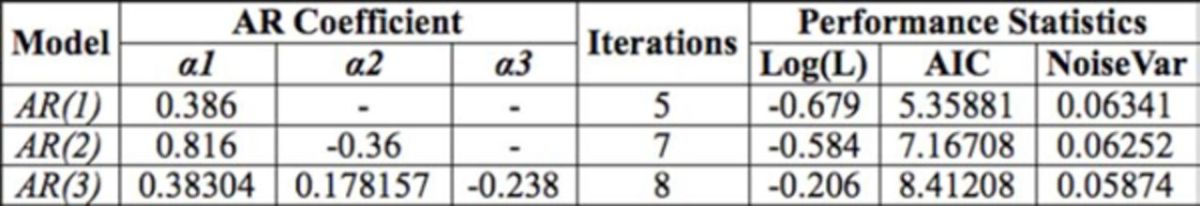
Parameter estimates and model evaluation statistics for the stochastic autoregressive model (AR) in respect to model order in modelling EIL variables.

**Figure 4.**
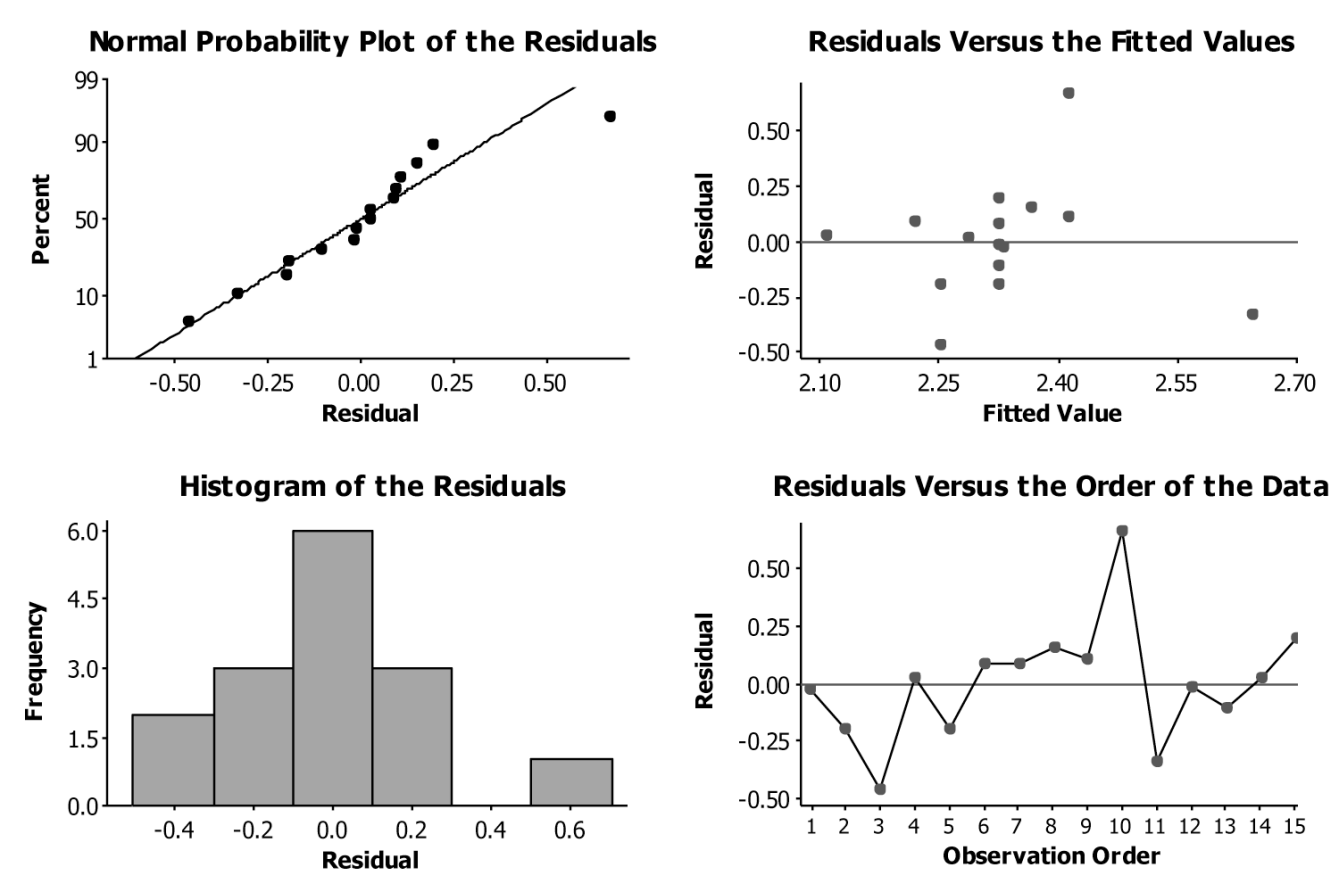
Residual plots and forecasting performance of the *AR(1)* model in describing seasonal trends of the EIL. High quality figures are available online.

Figure 4 illustrates the model performance of the *AR(1)* model in describing the seasonal trends of the EIL time series. According to the normal probability plot, the generated histograms, and the residual error plots, it is observable that the data are normally distributed, and therefore the selected *AR(1)* model describes with high accuracy most observations.

In a real time context, this model can be projected over the future (i.e., based on the autoregressive parameter values) and provide means to evaluate the profitability (or not) of certain varieties (or crops). For example, it is quite obvious that new varieties having higher EILs are advantageous compared to regular ones and are worth being planted in more areas. Furthermore, because selling of the crop is nearly always done shortly after harvest, while the EIL is applied earlier, the current long term analysis can be used to inform the necessity of short-term decision making using the predictions to gain threshold realities of the following year.

Figure 5 generates the predictions of the EIL values according to the applied autoregressive model having order one ( *AR(1)* ). Forecasts for 2010–2015 ranged from 2.33 to 2.41 individuals per sampling unit. These values represent a threshold that is in reasonable limits to justify future control actions taken by farmers. In other words, according to the applied model and the respective forecasts, high quality peach products (i.e., composts) can be produced for the next five years in the current frame of IPM and supply markets.

Moreover, Figure 5 also presents the generated model predictions according to the autoregressive parameter estimate of the *AR(1)* model. Simulated data are generally in reasonable limits, and forecasts matched over the years 2011 and 2012 were quite close to observed values.

The most related work, which deals with uncertainty and variability in the variables that determine the EIL, including crop market value, is that of Peterson and Hunt (2003) . Particularly, they have considered each EIL parameter separately and their probability distributions at a fixed point, and then propagated them into the output of the EIL model. Moreover, based on Monte Carlo simulations, they generated for each of the EIL parameters specific distributions, most of them having kurtosis and positive skewness (e.g., lognormal), and defined a probabilistic economic injury Level (PEIL).

In this work, however, we considered the EIL as a time process that is captured by a typical-normal joint distribution and without bootstrapping. Moreover, the approach differentiates conceptually, considering that we are interested in performing nonlinear predictions rather than estimating the degree of EIL uncertainty associated with the type of distribution and mean percentiles.

### Conclusion

Although economic-threshold models are deterministic in nature and either contain or are linked, to some extent, to population-dynamic models ( Dennis et al. 1986 ), they take the form of prediction models by including variables that express random behaviour in very few studies ( Peterson and Hunt 2003 ). However, this work handled the EIL’s concept as a dynamic stochastic process that is evolving in time and makes efforts to describe its behaviour based on linear autoregression models having different orders.

By this context the EIL formula is used as a deterministic skeleton, which was used to generate different outcomes according to yearly fluctuations of the economic variables. Since alterations on yearly price commodities of crop products mostly behave randomly, they also result in non-deterministic behaviour of the EIL. By this context, a model can be fitted on EIL serial data to describe the stochastic process and make forthcoming predictions.

To date, there is no dynamic EIL reported in the literature that incorporates yearly stochastic trends of the economic variables. In addi-addition, most procedures are focusing on the estimation of the yield function, or further proceed on the estimation of constant EILs, and the probabilistic EIL differs conceptually in comparison to the time series approach that was followed ( Pedigo et al. 1986 ; Onstad 1987 ; Pedigo 1995 ; Peterson and Hunt 2003 ; Moschos 2005 )

From a biological standpoint, although the construction of the damage function is a very difficult task and a prerequisite of estimating EILs, the multidisciplinary aspect of the EIL concept enables the evaluation of economic variables ( Southwood and Norton 1973 ; Plant 1986 ; Pedigo 1995 ; Damos and Savopoulou-Soultani 2012 ).

The issue of temporal EIL modelling may be much more complicated, considering that biological parameters are also affected temporally and spatially. Because in most cases insect feeding behaviour and development are temperature driven ( Logan et al. 1976 ; Samietz et al. 2007 ), environmental noise can modify the damage function. Additionally, other factors that affect the damage function and EIL are time delays on insect development and injury rates in respect to host performance ( Higley and Pedigo1993 ). Nevertheless, it is feasible for a given species and cultivation to construct the damage function, estimate the parameter *D* , and proceed to estimation of the economic variables.

Thus, for a regular presence of economically important pests in specific cultivation regions and for a given pesticide efficacy ( *K* ), the EIL is strongly governed by the cost of management ( *C* ) and the commodity value ( *V* ). In other words, different combinations among the random economic variables affect the respective EIL, and therefore the proposed stochastic approach in modelling EIL levels is useful in predicting future pest and crop specific economic threshold levels.

Finally, applying multivariate stochastic models can incorporate more potential variables and increase prediction capability of EILs and adoption of IPM systems towards sustainable agriculture.

## Abbreviations

EIL: economic injury level

## References

[R1] Agra CEAS . 2002 . Integrated Crop Management Systems in the EU . Amended Final Report for European Commission, DG Environment .

[R2] Akaike H . 1974 . A new look at the statistical model identification . IEEE Transactions on Automatic Control 19 ( 6 ): 716 – 723 .

[R3] Altieri MA Nicholls CI . 2000 . Agroecology in action. Indigenous and modern approaches to IPM in Latin America . ESPM Division of Insect Biology, University of California, Berkeley , USA . Available online: www.nature.berkeley.edu/~miguel-alt/indigenous_and_modern_approaches.html

[R4] Boller EF Avilla J Jörg E Malavolta C Wijnands F Esbjerg P . 2004 . Integrated Production: Principles and Technical Guidelines , 3rd edition. IOBC-WPRS. Available online: www.iobc.ch/iobc_bas.pdf

[R5] Buntin GD . 1996 . Economic thresholds for insect management . In: Higley LG, Pedigo LP, Editors. Economic thresholds for integrated pest management . pp. 128 – 147 . University of Nebraska Press .

[R6] Brockwell PJ Davis RA . 1996 . Time series: theory and methods , second edition. Springer-Verlag .

[R7] Cross JV Dickler E . 1994 . Guidelines for Integrated Production of pome fruits in Europe: IOBC Technical Guideline III. IOBC/WPRS Bulletin 17 : 1 – 8 . International Organization for Biological and Integrated Control of Noxious Animals and Plants .

[R8] Damos P Savopoulou-Soultani M . 2008 . Development and validation of models in forecasting the seasonal emergency and population dynamics of the peach twig borer *Anarsia lineatella* (Lepidoptera: Gelechiidae) in northern Greece . In: Proceedings of XXIII international congress of entomology , 6 – 12 July, Ed. by ICE Org. Comm., Durban, South Africa, ICE section 1, 267, Abstract No. 414.

[R9] Damos P Savopoulou-Soultani M . 2009 . Population dynamics of *Anarsia lineatella* (Lep: Gelechiidae) in relation to crop damage and development of Economic Injury Levels . J. Appl. Entomol. 134 : 105 – 115 .

[R10] Damos P Savopoulou-Soultani M . 2010 . Development and statistical evaluation of models in forecasting major lepidopterous peach pest complex for integrated pest management programs . Crop Prot . 29 , 1190 – 1199 .

[R11] Damos P Savopoulou-Soultani M . 2012 . Microlepidoptera of Economic Significance in Fruit Production: Challenges, Constrains and Future Perspectives of Integrated Pest Management . In: Cauterruccia L, Editor. Moths: Types, Ecological Significance and Control . Nova Science Publications .

[R12] Damos P Rigas A Savopoulou-Soultani M . 2011 . Application of Markov Chains and Brownian motion models on Insect Ecology . In: Earnshaw RC, Riley EM. Brownian Motion: Theory, Modelling and Applications . pp. 71 – 104 . Nova Science Publications .

[R13] Dennis B Kemp PW Beckwith C . 1986 . Stochastic Model of Insect: Estimation and Testing . Env. Entomol. 15 : 540 – 546 .

[R14] Dent D . 1994 . Integrated Pest Management . Chapman and Hall .

[R15] Dickler E Schäfermeyer S . 1991 . General principles, guidelines and standards for integrated production of pome fruit in Europe and procedures for endorsement of national and regional guidelines and standards . OILB Bulletin SROP1991/XI/3.

[R16] European Commission . 2009a . Directive of 21 October 2009 concerning the sustainable use of pesticides (2009/128/EC) . European Commission .

[R17] European Commission . 2009b . Final Report Development of guidance for establishing Integrated pests management (IPM) principles . European Commission .

[R18] Ehler L.E. . 2006 . Perspective integrated pest management (IPM): definition, historical development and implementation, and the other IPM . Pest Manag. Sci . 62 : 787 – 789 . 16786545 10.1002/ps.1247

[R19] Grunwald GK Hyndman RJ Tedesco L Tweedie RL . 2000 . Non-Gaussian conditional linear AR(1) models . Australian and New Zealand Journal of Statistics 42 : 479 – 495 .

[R20] Higley G Pedigo LP . 1993 . Environmental aspects of insect pest management. Economic injury level concepts and their use in sustaining environmental quality . Agric. Ecosyst. Environ . 46 : 233 – 243 .

[R21] Higley LG Pedigo LP , Editors. 1996 . The EIL concept. In: Economic thresholds for integrated pest management . University of Nebraska Press .

[R22] Hoyt SC Burts EC . 1974 . Integrated Control of Fruit Pests . Ann. Rev. Entomol . 19 : 231 – 252 .

[R23] Ifoulis A Savopoulou-Soultani M . 2006 . Developing the optimum sample size and multistage sampling plans for *Lobesia botrana* (Lepidoptera: Tortricidae) larval infestation and injury in northern Greece . J. Econ. Entomol . 99 : 1890 – 1898 . 17066827 10.1603/0022-0493-99.5.1890

[R24] IPM Europe 2000 . For the harmonisation of European support to developing countries in the use of IPM to improve agricultural sustainability . IPM Europe .

[R25] Logan JA Wollkind DJ Hoyt SC Tanigoshi LK . 1976 . An analytic model for description of temperature dependent rate phenomena in arthropods . Environ. Entomol . 5 : 1133 – 1140 .

[R26] Moschos T . 2005 . Yield loss quantification ad assessment of economic injury level for the anthophagus generation of the European grapevine moth *Lobesia botrana* Den. et Schiff. (Lepidoptera: Tortricidae) . Int. J. Pest. Manag . 51 : 81 – 89 .

[R27] Onstad DW . 1987 . Calculation of economic-injury levels and economic thresholds for pest management . J. Econ. Entomol . 80 : 297 – 303 .

[R28] Pedigo LP . 1995 . Closing the gap between IPM theory and practice . J. Agric. Entomol. 12 : 171 – 181 .

[R29] Pedigo LP Hutchins SH Higley LG . 1986 . Economic injury levels in theory and practice . Annu. Rev. Entomol . 31 : 341 – 368 .

[R30] Peterson RKD Hunt TE . 2003 . The probabilistic economic injury level: incorporating uncertainty into pest management decisionmaking . J. Econ. Entomol . 96 : 536 – 542 . 12852585 10.1603/0022-0493-96.3.536

[R31] Plant RE . 1986 . Uncertainty and economic threshold . J. Econ. Entomol . 79 : 1 – 6 .

[R32] Samietz J Graf B Höhn H Schaub L Höpli H . 2007 . Phenology modelling of major insect pests in fruit orchards from biological basics to decision support: the forecasting tool SOPRA . Bulletin OEPP/EPPO Bulletin 37 : 255 – 260 .

[R33] Southwood TRE Norton GA . 1973 . Economic aspects of pest management strategies and decisions . Ecol. Soc. Aust. Mem . 1 : 168 – 184 .

